# A Case of Atraumatic and Non-obstetric Vulvar Hematoma from Contralateral Internal Iliac Artery Rupture

**DOI:** 10.5811/cpcem.42023

**Published:** 2025-07-24

**Authors:** Roger Raveiro, Moshe Bengio, Justin Sharp, Geoffrey Lindblad, Danial Mir, Sean Serio

**Affiliations:** * HCA Florida Aventura Hospital, Department of Emergency Medicine, Aventura, Florida; † Baptist Health South Florida, Department of Emergency Medicine, Coral Gables, Florida; ‡ Florida Atlantic University, Charles E. Schmidt College of Medicine, Department of Emergency Medical Services, Boca Raton, Florida; § HCA Florida Aventura Hospital, Department of Radiology, Aventura, Florida

**Keywords:** Hematoma, Vulva injuries, Shock, Hemorrhagic, Arterial Rupture, Iliac Artery / injuries, Embolization, Therapeutic, Radiology, Interventional, Emergency Medicine, Gynecology

## Abstract

**Case Presentation:**

An 18-year-old female, gravida 0, para 0, with no significant past medical history presented with spontaneous left vulvar hematoma that started two hours prior to arrival. History also revealed amenorrhea for the past nine months, menorrhagia three days ago, and oral contraceptive use. Her vitals demonstrated tachycardia to 130s beats per minute but otherwise were normal, consistent with an early stage of hemorrhagic shock. Physical exam was remarkable for significant left labia majora hematoma with active hemorrhage on computed tomography from the right internal iliac artery. She underwent emergent gelfoam embolization with interventional radiology and subsequent hematoma evacuation with an obstetrician gynecologist.

**Discussion:**

Etiologies of vulvar hematomas fall within two categories: obstetric or non-obstetric. In rare circumstances, hematomas that lack evidence of obstetric or traumatic events are presumed to be of spontaneous artery rupture origin. Vulvar hematomas are a clinical diagnosis but can be challenging. The hallmark symptom is moderate to severe pain that is usually in the perineum but can be in the groin, abdominal and/or buttock regions depending on the size and location of the hematoma. A proper history and physical exam are essential to rule out the differential diagnoses such as vulvar varicosities, folliculitis, Bartholin gland cysts/abscesses or vulvar cancer. Management of vulvar hematomas is not well defined. Ultimately, clinical decision should be based on degree of hemodynamic stability, size of the hematoma, rate of expansion, risk or presence of pressure necrosis, urologic symptoms and presence of unremitting pain. To date, there are three reported spontaneous vulvar hematomas due to pudendal artery rupture and one due to internal iliac artery rupture. To the best of our knowledge, our case represents the second reported account of non-obstetric, non-traumatic spontaneous vulvar hematoma due to internal iliac artery rupture and the first reported account where the resulting hematoma was contralateral to the affected artery.

## CASE PRESENTATION

An 18-year-old female, gravida 0, para 0, with no significant past medical history presented with spontaneous left vulvar hematoma that started two hours prior to arrival. She reported amenorrhea for the past nine months, however, began experiencing menorrhagia three days ago. She reported being on an oral contraceptive, uncertain of the type. She denied recent trauma or sexual activity.

**Image 1:**
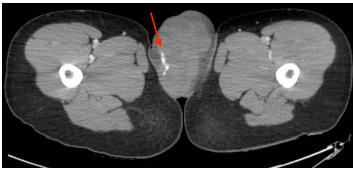
Computed tomography with angiography of the pelvis in axial view demonstrating arterial extravasation (red arrow) with surrounding hematoma formation extending into left vulva.

Initial vitals were as follows: temperature 36.7 °C, pulse ox 98% O_2_ on room air, blood pressure 126/91 millimeters of mercury (mm Hg), heart rate 138 beats per minute, and respiratory rate 19 breaths per minute, consistent with an early stage of hemorrhagic shock, according to the American College of Surgeons Advanced Trauma Life Support hemorrhagic shock classification system. Physical exam was remarkable for significant left labia majora hematoma without active external hemorrhage. Right vulva was normal in appearance. Complete blood count, comprehensive metabolic panel, and coagulation studies were unremarkable. Computed tomography of the pelvis with intravenous (IV) contrast showed left vulva hematoma measuring 12.4 cm x 6.8 cm x 7.0 cm with active arterial hemorrhage from the right internal iliac artery (image 1).

**Image 2:**
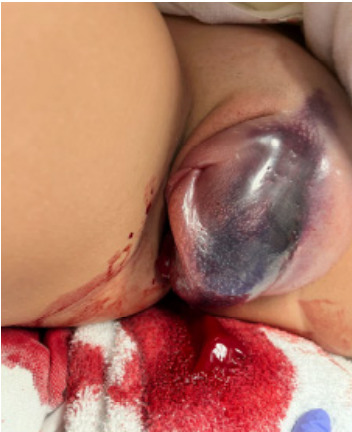
Lithotomy view demonstrates large, slowly extravasating vulvar hematoma with vulvar ecchymosis. This figure clarifies that this is a left vulvar hematoma with deviation of the vulva to the right. Not visible is four cm skin breakdown of the left vaginal wall.

While pending interventional radiology recommendations, repeat examination revealed the hematoma with area of skin breakdown and external hemorrhage (image 2). Patient’s tachycardia remained unchanged; mean arterial pressure dropped from 103 mm Hg to 72 mm Hg. Repeat labs showed hemoglobin dropped from 14.3 grams per deciliter (g/dL) to 11.9 g/dL (reference range: 11.2 – 15.7 g/dL) (likely secondary to hemorrhage and/or dilutional effect from the one liter normal saline IV bolus). One unit of uncrossmatched packed red blood cells and 1 g tranexamic acid IV administered while in the emergency department.

**Image 3:**
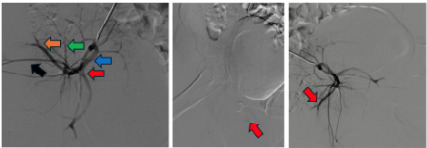
Left: Digital subtraction angiogram (DSA) of normal right iliac artery for reference: red = internal iliac anterior division, blue = internal iliac posterior division, green = iliolumbar artery, orange = lateral sacral artery, black = superior gluteal artery. Middle: right internal iliac DSA with focal extravasation: Red arrow points to a subselective catheterization of right internal iliac artery anterior division with a small focus of extravasation. Right: right internal iliac DSA post embolization: Red arrow points to selective catheterization of right internal iliac artery with DSA following gel foam embolization demonstrating truncation and pruning of vessel.

The patient was taken for emergent right internal iliac gelfoam embolization (image 3). Obstetrics and gynecology was consulted for right vulvar hematoma evacuation, which occurred five hours post embolization. The patient had resolution of hemorrhage and her hemoglobin was approximately 10 g/dL post-embolization and mid 9s g/dL on next morning labs with an upward trend thereafter. She was discharged home on post operative day five after achieving tolerable pain control.

## DISCUSSION

The vulva is the external part of the female genitalia consisting of the labia, clitoris and vestibule. It is located in the anterior half of the perineum, also known as the urogenital triangle. Arterial supply to this region is primarily carried out by terminal branches of the internal pudendal artery which comes off the anterior division of the internal iliac artery. Vulvar hematomas most commonly arise from insult to these terminal branches, frequently the posterior labial branch, but can rarely arise from the pudendal artery as well as the internal iliac artery as in our case. The superficial perineal pouch, the potential space formed by the perineal membrane (formerly urogenital diaphragm) and Colles fascia, allows for isolated vulvar hematomas to collect as fluctuant masses that expand outwardly towards the skin and can reach sizes greater than 15 cm.^1^,^2^

*CPC-EM Capsule*

What do we already know about this clinical entity?

*Vulvar hematomas are generally due to obstetric or traumatic causes, but, rarely, spontaneous local artery rupture can occur*.

What makes this presentation of disease reportable?

*This presentation is the only reported case of a spontaneous vulvar hematoma secondary to a contralateral internal iliac artery rupture*.

What is the major learning point?

*Vulvar hematomas are a clinical diagnosis but can be challenging. A proper history and physical exam are essential. Management is not well defined*.

How might this improve emergency medicine practice?

*Exposure to rare cases like this one allows for emergency medicine physicians to broaden their knowledge base and differential diagnoses*.

Etiologies of vulvar hematomas fall within two categories: obstetric or non-obstetric. Obstetric related hematomas are either due to direct injury during labor such as from instrumentation use, laceration repairs and episiotomies, or indirect injury such as from excessive stretching of birth canal during vaginal deliveries. Risk factors include primiparity, coagulopathies, use of anticoagulants, vulvovaginal varicosities, macrosomia, hypertensive disorders of pregnancy and prolonged second stage of labor.^3^–^5^

Non-obstetric related hematomas are mostly attributed to trauma to the perineum. Insults include but are not limited to vulva surgery, foreign body insertion, saddle injury, sexual assault as well as consensual coitus.^6^,^7^ Age is a significant risk factor for trauma-related vulvar hematomas. Pre-pubertal children and adolescents have decreased vulval fat, notably in the labia majora, which normally acts as a protective barrier for the underlying structures in healthy adult women. Similarly, hypoestrogenic postmenopausal women are more susceptible to arterial injury from loss of vulvar elasticity and vaginal atrophy.^2^

In rare circumstances, hematomas that lack evidence of obstetric or traumatic events are presumed to be of spontaneous artery rupture origin, mainly the internal pudendal or the internal iliac arteries. Rupture of iliac artery is most associated with aneurysms secondary to atherosclerotic disease but can also be caused by coagulopathies. Rarely the aneurysms can be due to connective tissue disease or infections. In cases where traumatic events do not result in immediate arterial ruptures, pseudoaneurysms can form that may lead to spontaneous vulvar hematomas on a later date. This is most associated with the pudendal artery rather than the internal iliac artery.^1^,^8^

Vulvar hematomas are a clinical diagnosis but can be challenging. The hallmark symptom is moderate to severe pain that is usually in the perineum but can be in the groin, abdominal and/or buttock regions depending on the size and location of the hematoma. Additionally, neurologic and urologic symptoms, from compression of the nerve roots and bladder or urethra respectively, can be observed. Complications can include infection, pressure necrosis and hemodynamic instability.^1^,^8^,^9^ A proper history and physical exam are essential to rule out the differential diagnoses such as vulvar varicosities, folliculitis, Bartholin gland cysts/abscesses or vulvar cancer.

Management of vulvar hematomas is not well defined. Small and stable hematomas can be treated expectantly and conservatively with local compression, ice packs and analgesics, as most will resolve on their own. The exact point at which a hematoma would benefit from operative or selective artery embolization management has not been established. Among the reported cases where invasive treatment was pursued, hematoma sizes varied greatly ranging anywhere from 7 cm to 15 cm and were present with other serious complications such as hemodynamic instability and/or pressure necrosis.^9^–^11^ Ultimately, clinical decision should be based on degree of hemodynamic stability, size of the hematoma, rate of expansion, risk or presence of pressure necrosis, urologic symptoms and presence of unremitting pain.^2^,^9^ Important to note, a retrospective study found that conservative treatment increased risks of complications leading to longer hospital stays, infections and need for transfusions when compared to operative management.^12^

Although a definitive explanation as to how a bleed originating from the right internal iliac artery resulted in a left vulvar hematoma could not be established, we surmise the complex network of vascular anastomoses within the pelvic cavity, in conjunction with the continuity of the fascial planes in this region, allowed for extravasated blood to cross the midline via flow through established vascular networks.^13^–^15^

The majority of vulvar hematomas are obstetric in nature. Non-obstetric hematomas have an incidence of 3.7% and make up 0.8% of all gynecological emergencies. Of these, even a smaller percentage constitutes spontaneous etiology.^16^ To date, there are three reported spontaneous vulvar hematomas due to pudendal artery rupture and one due to internal iliac artery rupture.^1^,^10^,^17^,^18^ To the best of our knowledge, our case represents the second reported account of non-obstetric, non-traumatic spontaneous vulvar hematoma due to internal iliac artery rupture and the first reported account where the resulting hematoma was contralateral to the affected artery.
